# CREB-binding protein plays key roles in juvenile hormone action in the red flour beetle*, Tribolium Castaneum*

**DOI:** 10.1038/s41598-018-19667-6

**Published:** 2018-01-23

**Authors:** Jingjing Xu, Amit Roy, Subba Reddy Palli

**Affiliations:** 1 Department of Entomology, College of Agriculture, Food and Environment University of Kentucky, Lexington, KY 40546 USA; 2 Faculty of Forestry and Wood Sciences, EXTEMIT-K, Czech University of Life Sciences, Kamýcká 1176, Prague 6, Suchdol 165 21 Czech Republic

## Abstract

Juvenile hormones (JH) and ecdysteroids regulate many biological and metabolic processes. CREB-binding protein (CBP) is a transcriptional co-regulator with histone acetyltransferase (HAT) activity. Therefore, CBP is involved in activation of many transcription factors that regulate expression of genes associated with postembryonic development in insects. However, the function of CBP in JH action in insects is not well understood. Hence, we studied the role of CBP in JH action in the red flour beetle, *Tribolium castaneum* and the *Tribolium* cell line. CBP knockdown caused a decrease in JH induction of genes, Kr-h1, 4EBP and G13402 in *T. castaneum* larvae, adults and TcA cells whereas, Trichostatin A [TSA, a histone deacetylase (HDAC) inhibitor] induced the expression of these JH-response genes. Western blot analysis with specific antibodies revealed the requirement of CBP for the acetylation of H3K18 and H3K27 in both *T. castaneum* and TcA cells. Chromatin immunoprecipitation (Chip) assays showed the importance of CBP-mediated acetylation of H3K27 for JH induction of Kr-h1, 4EBP, and G13402 in TcA cells. These data suggest that CBP plays an important role in JH action in the model insect, *T.castaneum*.

## Introduction

Juvenile hormones (JH) regulate many aspects of insect’s life cycle including reproduction and development. Recently, a bHLH transcription factor methoprene-tolerant (Met) was identified as a JH receptor in the fruit fly, *Drosophila melanogaster* and other model insects including *Bombyx mori*, *Aedes aegypti, Blattella germanica* and *Tribolium castaneum* [see^1,2^ for review]. Moreover, current studies on JH signaling pathway revealed mechanisms of JH action as well as its cross-talk with 20-hydroxyecdysone (20E), insulin signaling and WNT pathways^3–11^. Most of these studies focused on understanding how JH regulates gene expression in the model insects including the fruit fly, mosquito, red flour beetle, cockroach and silk moth. Hundreds of genes regulated by JH have been identified in these insects, and one gene consistently identified as an important player in JH action is krüppel homolog 1 (Kr-h1)^12–17^. The kr-h1 expression is regulated by both JH and 20-hydroxyecdysone^18,19^. The expression of kr-h1 is directly induced by JH through Met, steroid receptor coactivator (SRC) and juvenile hormone response elements (JHRE) present in the promoter region^14,20,21^. However, not much is known about the effect of epigenetics and post-translational modifications on JH action.

Epigenetic regulation and post-translational modification of proteins by acetylation, phosphorylation, and methylation regulate many cellular processes in living organisms. In the honey bee, DNA methylation plays important roles in the regulation of caste differentiation^22,23^ and memory processing^24^. The acetylation of histones by histone acetyltransferases (HATs) results in neutralization of lysine residues causing an increase in accessibility to promoters and gene expression. In *D*. *melanogaster*, lysine acetylation sites have been identified in the proteome using high-resolution mass spectrometry^25^. The sites of these modifications are highly conserved between humans and fruit flies. Furthermore, a study comparing lysine acetylation sites among fruit fly, human, nematode and zebrafish showed significantly higher conservation on the acetylated lysine residues than on the non-acetylated ones^25^. Genome-wide studies in *D. melanogaster* detected the presence phosphoacetylation as a general feature of enhancers and promoters^26^. In *Galleria mellonella*, histone acetylation has been shown to enhance transcription during insect metamorphosis, wounding and infection^27^.

Acetylation also plays important roles in the hormonal regulation of gene expression. In *D*. *melanogaster*, the epigenetic factors including Brahma (Brm, containing chromodomain and a HAT domain,) and CREB-binding protein (CBP, containing Bromodomain and HAT domain) were shown to play critical roles in initiating dendrite pruning. The HAT activity of CBP involved in acetylation of H3K27 is required for sox14 expression, which is an ecdysone response gene^28^. Acetylated H3K23 is localized to the promoters of Eip74EF and Eip75B, the 20E-induced transcription factors that play key roles in ecdysteroid action, and the acetylation levels of H3K23 correlate with the 20E induced expression of these genes. Here also, acetylation is promoted by nejire, the CBP homolog^29^. H3K122 acetylation is catalyzed by the co-activators, p300/CBP, and is induced by nuclear hormone receptor signaling^30^.

Besides acetylation of histones, the acetylated nuclear proteins such as transcription factors FoxO and P53 are also involved in regulation of gene expression^31–36^. Influence on the acetylation status of FoxO1 by Sirt2 in 3T3-L1 adipocytes enhances insulin-stimulated phosphorylation of FoxO1, which in turn regulates FoxO1 nuclear and cytosolic shuttling^34^. The acetylation of P53 influences its stability^37^, DNA binding activity^38^ and interaction with other proteins^39^. However, the role of acetylation in JH action is not known yet. In the current study, knockdown of CBP expression or application of TSA modified acetylation levels of histones in T*. castaneum* larvae, adults and TcA cells. Western blots and chromatin immune precipitation experiments showed that CBP and H3 acetylation play an important role in JH action.

## Results

### TcA cells respond to both Juvenile hormone and 20-hydroxyecdysone

**Figure 1:**
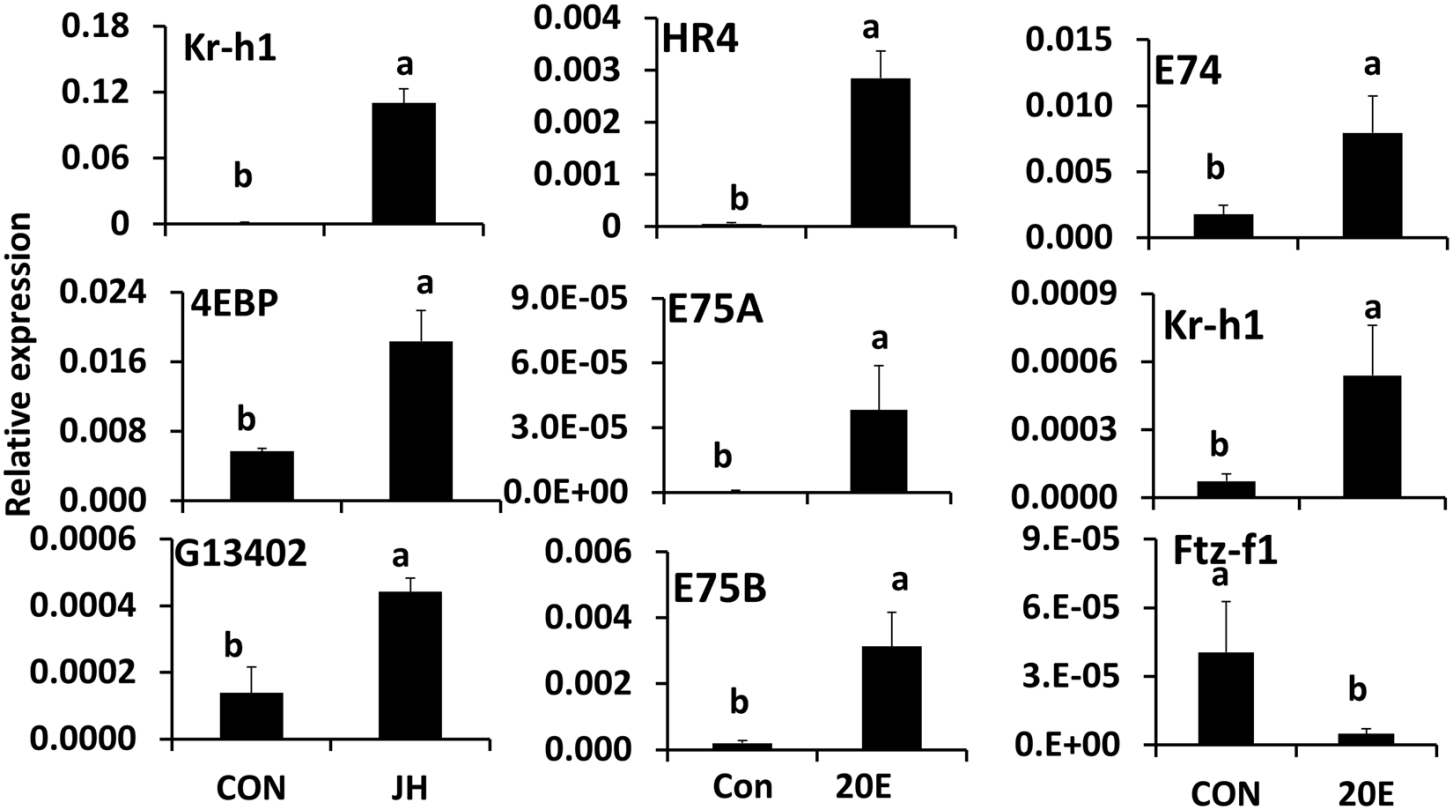
TcA cells respond to both juvenile hormone and 20-hydroxyecdysone. TcA cells respond to 10 µM JH III or 20E. Total RNA was isolated from 100,000 cells that were cultured in the medium containing either DMSO or JH III or 20E at a final concentration of 10 µM for 6 hr. Total RNA was converted to cDNA, and the relative levels of Kr-h1, 4EBP, G13402, HR4, E74, E75A, E75B and Ftz-f1 mRNA were determined by qRT-PCR using RP49 as a control. The data shown are the Mean + S.D. (n = 3). The numbers on the control Kr-h1 bar show the relative expression levels for this treatment.

The *Tribolium castaneum* cell line (TcA) has been developed from the *Tribolium castaneum* pupae and adult tissues^40^. To determine whether TcA cells respond to two major insect hormones, we tested JH III and 20E response in these cells. The TcA cells exposed to 10 µM JH III and showed an increase in Kr-h1, 4EBP and G13402 mRNA levels by 77.3, 3.2 and 3.2-fold respectively, when compared to their levels in cells treated with DMSO (Fig. 1). Similarly, exposure of these cells to 10 µM 20E induced the expression of HR4, Kr-h1, E74, E75A, and E75B and suppressed the expression of Ftz-f1 (Fig. 1). These data showed that TcA cells respond to both JH III and 20E.

### CBP functions in JH III action in the TcA cells

**Figure 2:**
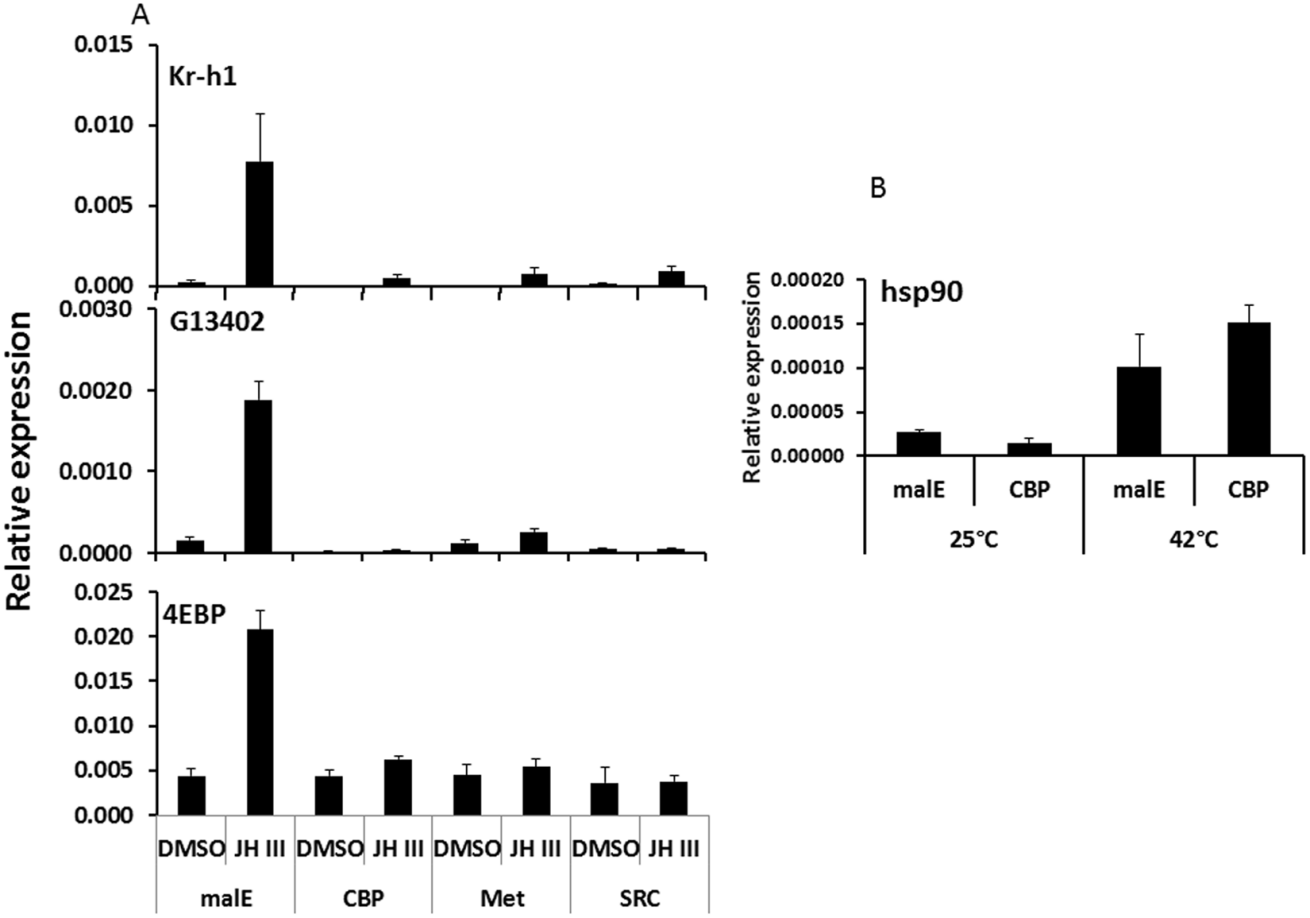
CBP is required for induction of JH-response genes in TcA cells. (**A**) CBP is required for JH induced gene expression. Total RNA was isolated from 100,000 cells exposed to malE, TcCBP, TcMet and TcSRC dsRNA for 72 hr. The cells were then exposed to 10 µM JH III for 6 hr. Total RNA was converted to cDNA, and the relative Kr-h1, 4EBP, and G13402 mRNA levels were determined by qRT-PCR using RP49 as a control.  The data shown are the Mean + S.D. (n = 3). (**B**) CBP is not required for gene expression induced by heat shock; Total RNA was isolated from 100,000 cells cultured with malE, TcCBP dsRNA for 72 hr followed by heat shock at 42 °C for 1hr. The relative levels of hsp90 mRNA were determined by qRT-PCR using RP49 as a control. The data shown are the Mean + S.D. (n = 3).

CBP is known to act as a co-activator in the expression of genes involved in 20E signal transduction. To test the function of CBP in JH III regulation of genes in TcA cells, the expression of CBP was knocked down in TcA cells by exposing these cells to CBP dsRNA in the medium for 72 hr. The cells exposed to dsRNA were treated with DMSO or 10 µm JH III and the total RNA isolated from these cells was used in qRT-PCR to determine the relative expression of JH-response genes. The TcA cells exposed to CBP dsRNA showed a 60% reduction in CBP mRNA levels when compared to the levels in control cells exposed to malE dsRNA (malE is coding for maltase in E. coli and there is no matching sequence found in *T. Castaneum* genome). All the three JH-response genes Kr-h1, 4EBP and G13402 tested were not induced by JH III in the cells exposed to CBP dsRNA. In contrast, the control cells exposed to malE dsRNA, showed an increase in Kr-h1, 4EBP and G13402 mRNA levels by 33.7, 11.8 and 4.8-fold upon JH III treatment (Fig. 2A). These data demonstrated the requirement of CBP in JH III induction of Kr-h1, 4EBP, and G13402 expression. Knockdown of JH receptor gene Met and co-activator, SRC also reduced the expression of the same genes in TcA cells (Fig. 2A). Interestingly, CBP is known to regulate gene expression through chromatin modification affecting the recruitment of proteins to promoters. To test whether CBP is involved in the expression of all genes, we tested expression of heat-shock gene, hsp90 in CBP knockdown cells. Heat-shock treatment of TcA cells was performed at 42 °C for one hour. Hsp90 gene was induced in cells exposed to 42 °C compared to the expression in cells exposed to 25 °C. Interestingly, the expression of this gene was also induced in the cells treated with CBP dsRNA to the same levels as in the control cells (Fig. 2B) suggesting that CBP is not required for induction of hsp90 gene in TcA cells.

### CBP is required for JH induction of gene expression in *T. castaneum* larvae and adults

**Figure 3:**
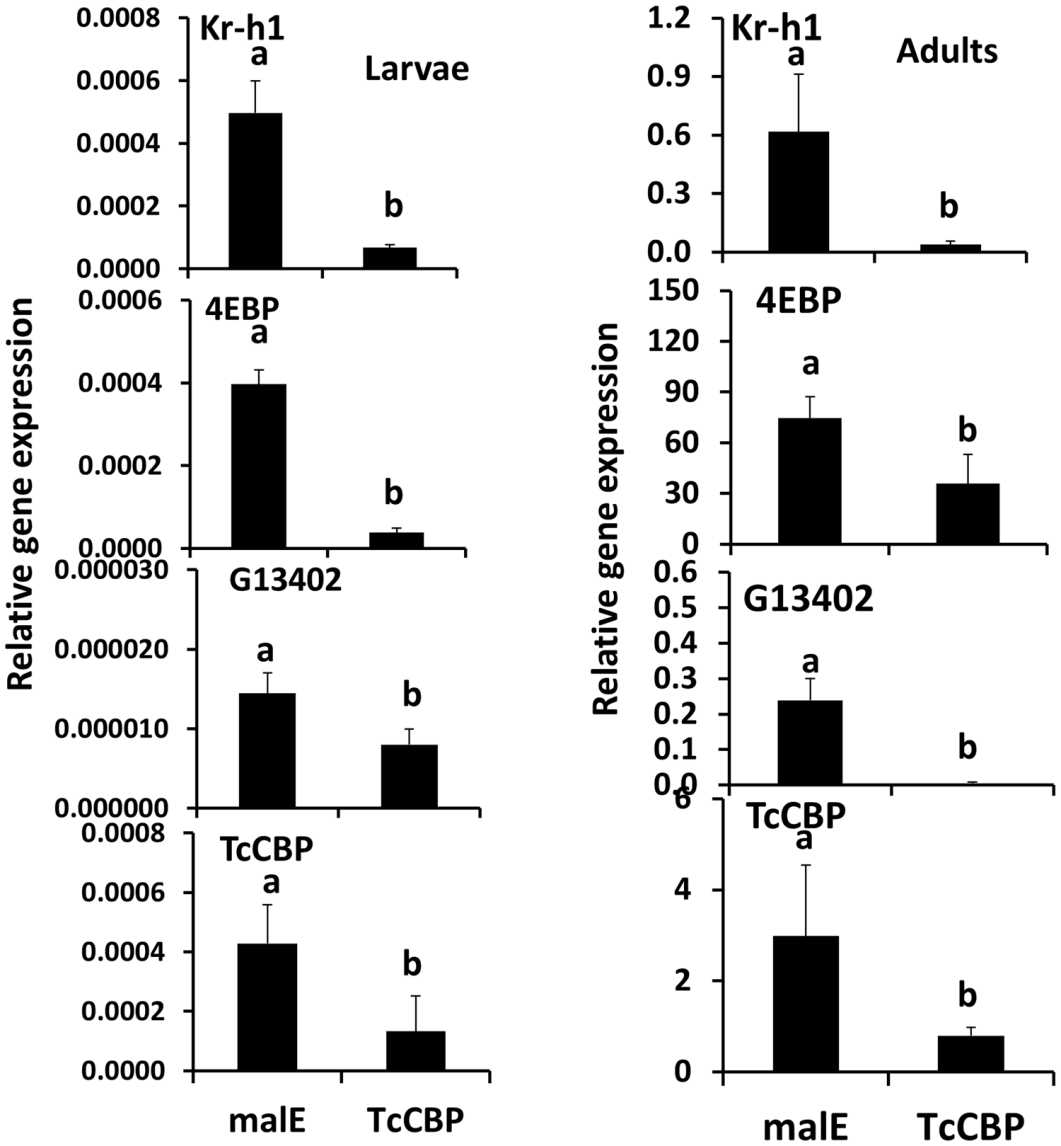
CBP is required for JH induction of genes in final instar larva (left panel) and newly emerged adults (right panel) of *T*. *castaneum*. CBP is required for JH induction of Kr-h1, 4EBP, and G13402 in day three adults or final instar larva. The newly emerged adults or the final instar larva were injected with either malE or CBP dsRNA. Seventy-two hours after injection 0.5 µl of 10 μM hydroprene was topically applied. Six hours after application, total RNA was isolated and used to quantify mRNA levels of Kr-h1, 4EBP, and G13402 by qRT-PCR using RP49 as a control. The data shown are the Mean + S.D. (n = 4).

To determine whether CBP is required for JH induced gene expression *in vivo*, TcCBP was knocked down by injection of TcCBP dsRNA into the newly ecdysed final instar larvae and newly emerged adults. Because the JH III levels are low in day 3 final instar larvae and newly emerged adults^41^, the JH analog hydroprene at 10 µm concentration was topically applied to these insects at 72 hr after injection of CBP or control malE dsRNA. At 6 hr after application of hydroprene, the total RNAs were isolated and used to determine relative mRNA levels using qRT-PCR. The mRNA levels of Kr-h1, 4EBP, G13402 and CBP significantly decreased by 93.8, 52.1, 98.0 and 80.2%, in the final instar larvae (Fig. 3) 83.2, 90.4, 44.7% and 70.1% respectively in day 3 adults (Fig. 3) injected with TcCBP dsRNA, when compared to their levels in control insects injected with malE dsRNA (*P* < 0.0001). These experiments showed that CBP is required for JH-induced gene expression *in vivo* as well.

### Histone deacetylase (HDAC) inhibitor, Trichostatin A, induces expression of JH-response genes

**Figure 4:**
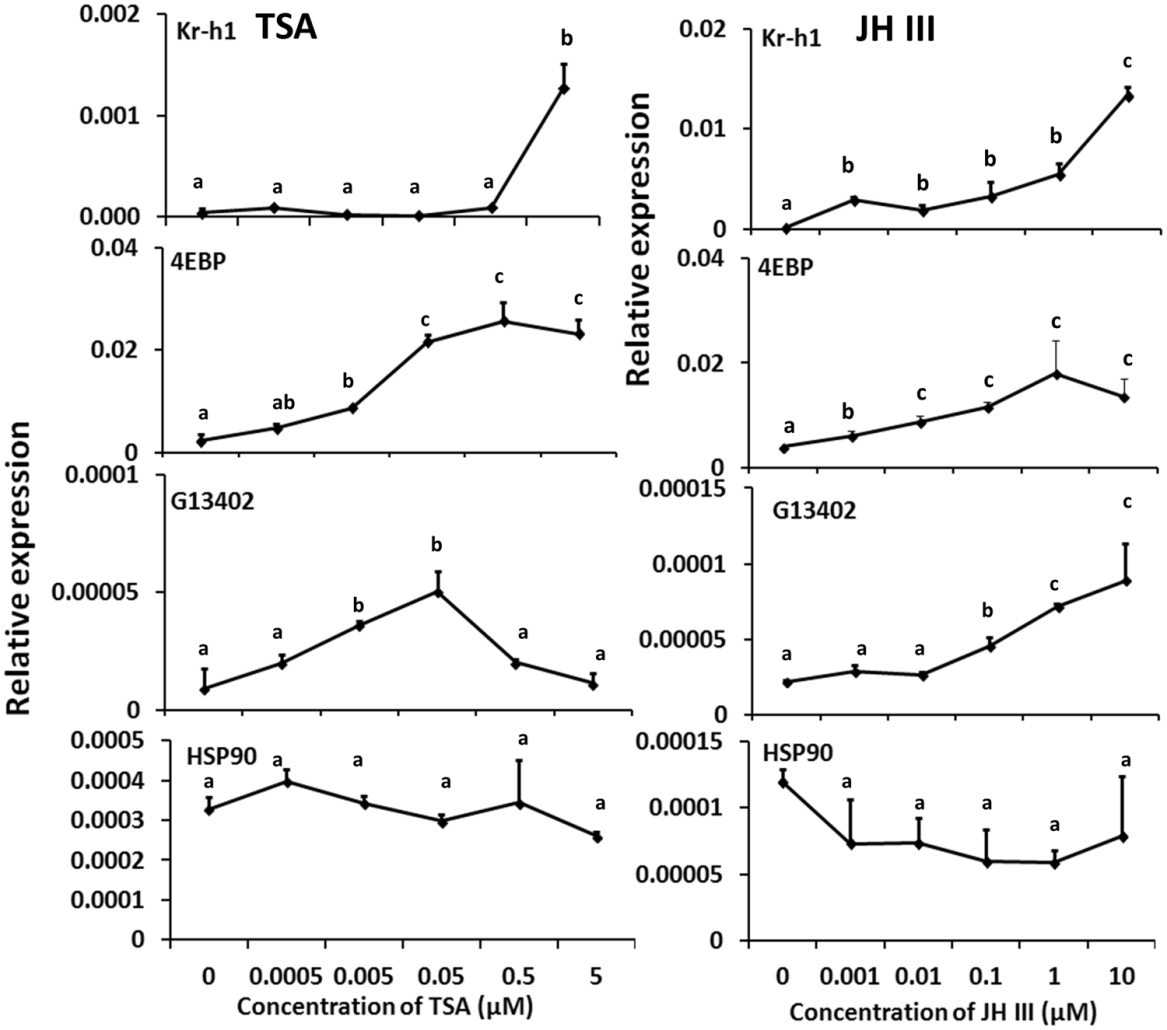
Trichostatin A (TSA, HDAC inhibitor, left panel) and JH III (right panel) induce Kr-h1, 4EBP and G13402 expression in a dose-dependent manner. TcA cells were cultured in the medium containing 0–5 μM TSA or 0–10 μM JH III for 6 hr. Total RNA was isolated from the treated cells and used to quantify mRNA levels of Kr-h1, 4EBP, G13402, and hsp90. The total RNA extraction and subsequent quantification of mRNA levels of Kr-h1, 4EBP, G13402, and hsp90 were performed as described in Fig. 1. The data shown are the Mean + S.D. (n = 3).

Since CBP is known to be involved in acetylation of proteins due to its HAT domain function. we hypothesized that HDACs might also influence JH action. To test this hypothesis, we used Trichostatin A (TSA), an inhibitor of HDAC. TcA cells were exposed to different doses of TSA for 6 hr and the mRNA levels of JH-response genes were quantified. The Kr-h1 mRNA levels increased in TcA cells exposed to 5 µM TSA (Fig. 4). Both 4EBP and G13402 mRNA levels began to increase in cells exposed to 0.005 µM TSA and reached the maximum levels in cells exposed to 0.05 µM TSA. While 4EBP mRNA levels remained high, the G13402 mRNA levels declined in cells exposed to 0.5 and 5 µM TSA. The cells exposed to JH III showed an increase in Kr-h1 mRNA levels beginning at 0.001 µM hormone and reached the maximum levels in cells exposed to 10 µM JH III (Fig. 4). In JH III exposed cells, the 4EBP mRNA levels began to increase at 0.001 µM JH III and reached the maximum levels in cells exposed to 1 µM JH III. The G13402 mRNA levels began to increase in cells exposed to 0.1 µM JH III and reached the maximum levels in cells exposed to 10 µM JH III. In control experiments, neither JH III nor TSA induced hsp90 gene.

### CBP does not directly affect expression of JH receptor, Met, and co-activator, SRC

**Figure 5:**
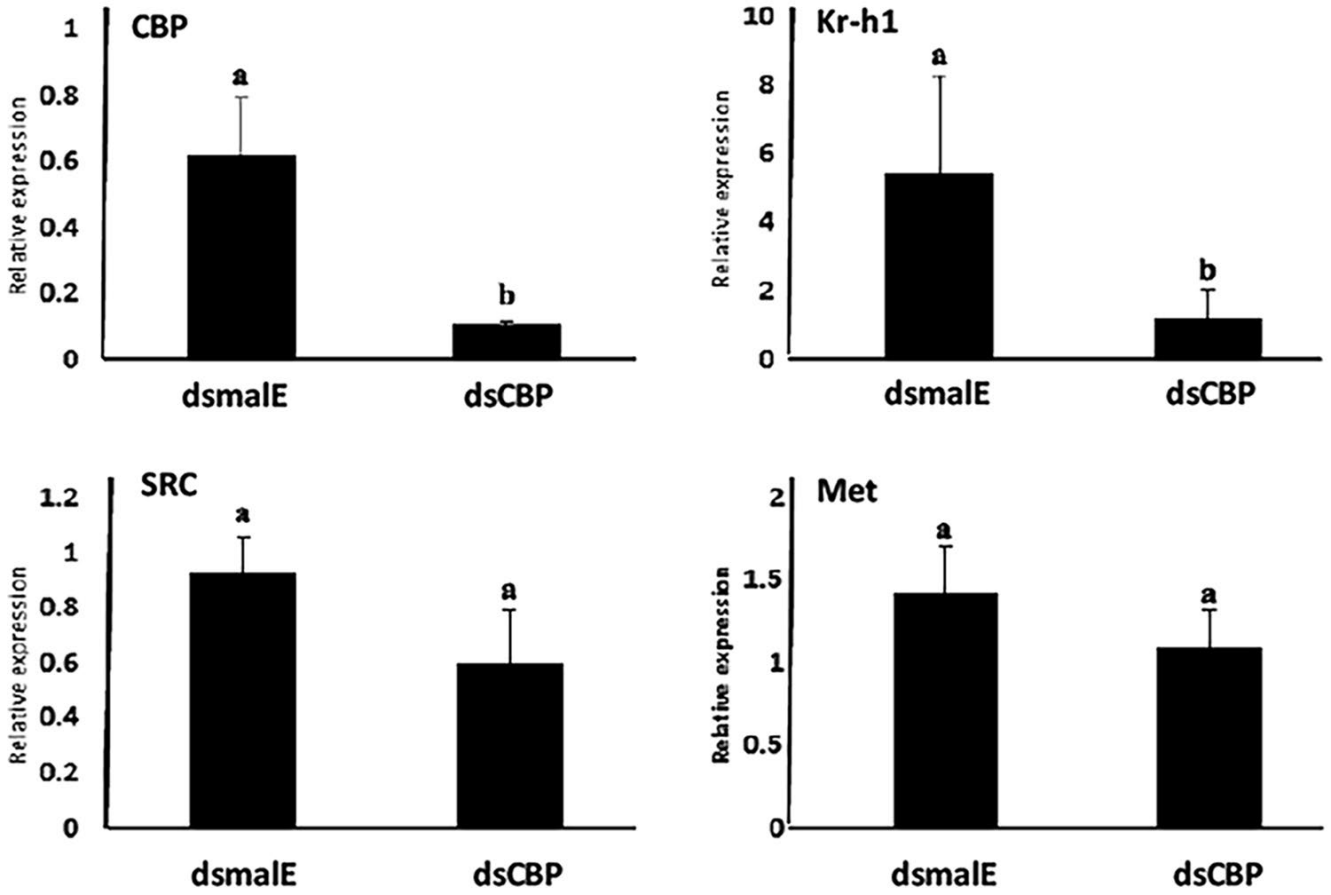
CBP is not required for expression of JH receptor Met and steroid receptor co-activator, SRC in TcA cells. Total RNA was isolated from 100,000 cells exposed to malE or TcCBP dsRNA for 72 hr. Total RNA was converted to cDNA, and the relative mRNA levels of CBP, Met, SRC and Kr-h1 were determined by qRT-PCR using RP49 as a control. The data shown are the Mean + S.D. (n = 3). (One-way ANOVA, letters represent significance at 95% CI).

To determine if the reduction in expression of JH-response genes in CBP-knockdown cells is due to direct action of CBP on the expression of JH receptor, Met and co-activator, SRC, we exposed TcA cells to dsCBP and determined relative mRNA levels of CBP, Kr-h1, Met, and SRC. As shown in Fig. 5, at 72 hr exposure to dsCBP, the CBP and Kr-h1 mRNA levels showed a significant reduction as expected. Interestingly, neither Met nor SRC mRNA levels showed a significant decrease in dsCBP exposed cells when compared to the control cells exposed to dsmalE.

### CBP modulates JH-response acting as a histone acetyltransferase (HAT)

**Figure 6:**
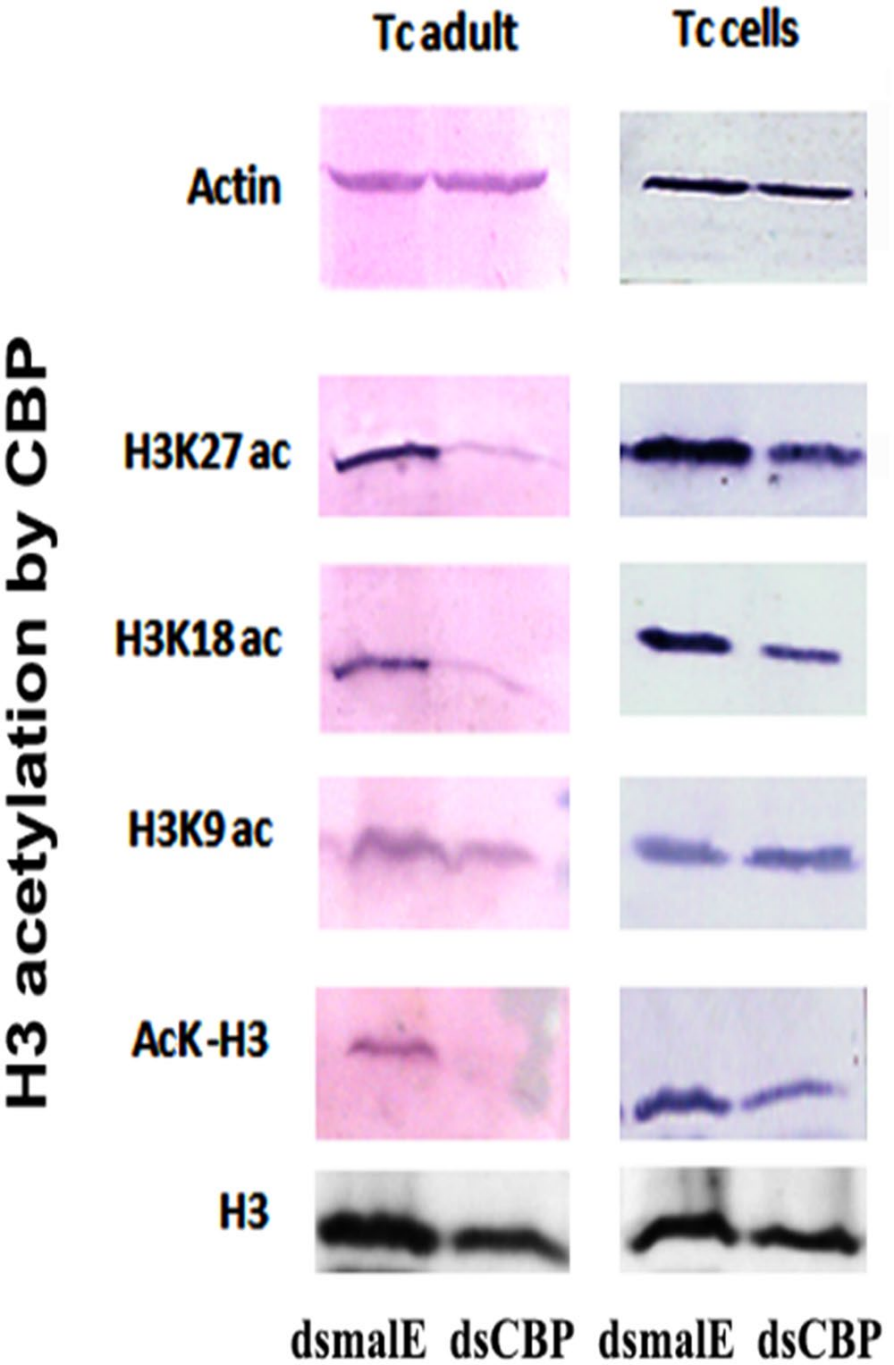
CBP is involved in the acetylation of H3 in *T. castaneum* adults and TcA cells. 50 µg total proteins extracted from the day 5 adults and TcA cells were separated by SDS-PAGE gels, transferred to the membrane and probed with acetyl-lysine, H3K9, H3K18 and H3K27 antibodies. The same concentration of proteins in parallel blots were probed with actin and H3 antibodies are shown on the top and bottom. The blots shown are cropped from the full-length blots presented in Supplementary Figure 1.

To determine if CBP modulates JH-response by acting as a histone acetyltransferase, acetylation status of three lysine residues H3K9, H3K18 and H3K27 was determined in TcA cells. When CBP was knocked down by culturing TcA cells in the presence of TcCBP dsRNA for 72 hr, the total ‘acetylated H3′ levels showed a decrease when compared to that in control cells as determined by Western blots using acetylated lysine antibodies (Fig. 6). The acetylation of both H3K18 and H3K27 but not the H3K9 showed a decrease in CBP knockdown cells and may have contributed to the differences in total acetylation of H3 protein observed (Fig. 6). These data suggest that CBP is involved in H3K18 and H3K27 acetylation in the TcA cells. A similar reduction in acetylation was observed *T. castaneum* adult tissues after knockdown of CBP by injection of dsRNA (Fig. 6).

### Chromatin Immunoprecipitation Assay

**Figure 7:**
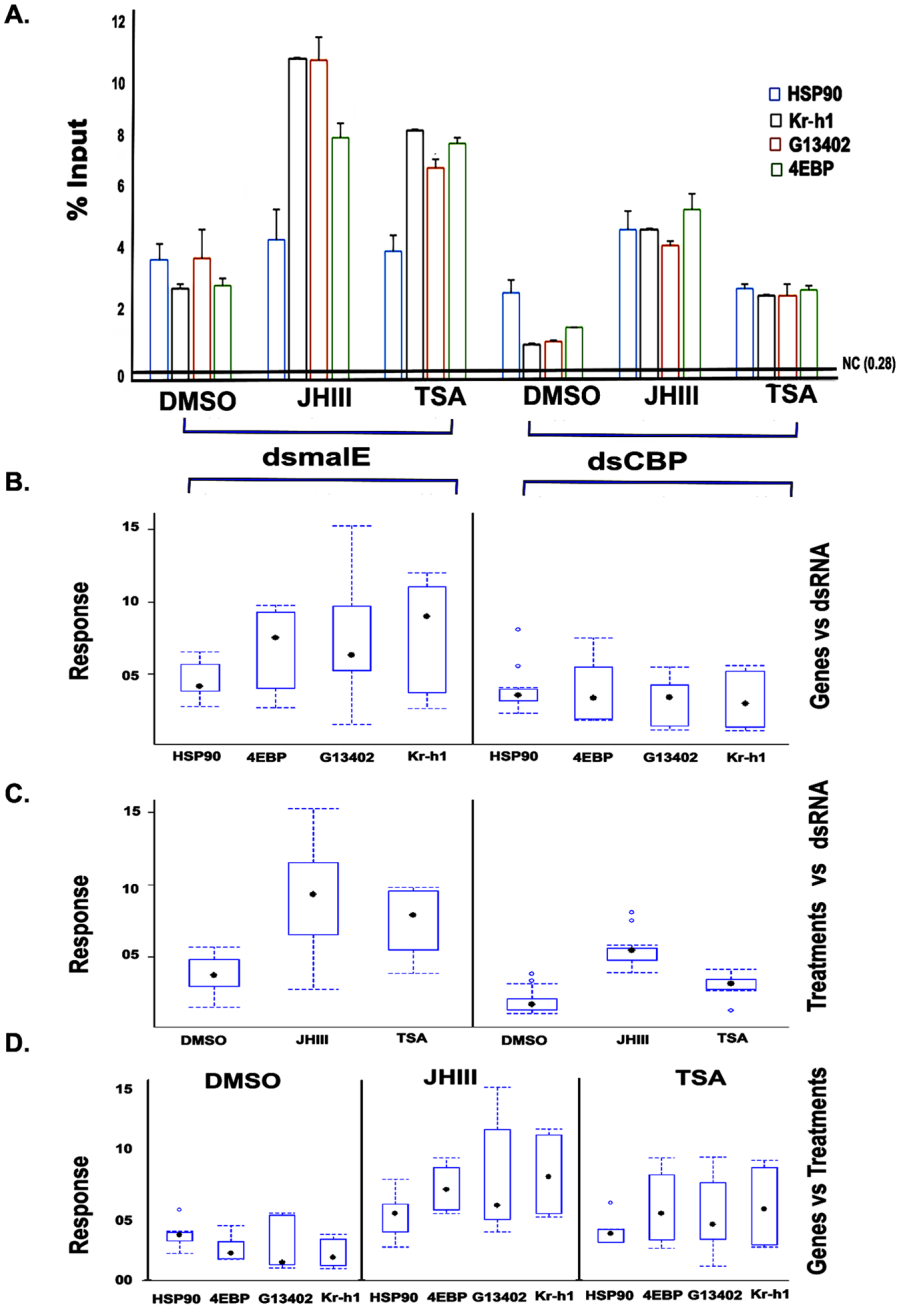
(**A**) Chip assay. dsCBP exposure decreased the localization of the acetylated H3K27 to Kr-h1, 4EBP, and G13402 promoters thus affected the enrichment of the gene promoters compare to the cells exposed to dsmalE. The cells were exposed to dsCBP or dsmalE and treated with DMSO, JH III, and TSA. The chromatin was cross-linked and precipitated with H3K27 antibodies. The DNA was amplified using primers targeting Kr-h1, 4EBP,G13402 or HSP90 gene promoters. The highest expression level in the negative control experiment (assay with IgG antibody) was indicated by a solid line and marked as NC. (**B–D**) Boxplots showing the results of statistical analysis represented in the tabular form in Table 2. Each interaction corresponded to an individual plot.

Chip assay using TcA cells and H3K27ac antibody showed a significantly enhanced enrichment of the promoter regions of all three JH response genes, Kr-h1, 4EBP and G13402 after JH III and TSA treatment when compared to control cells treated with DMSO (Fig. 7A, *P* < 0.001). The estimated fold change of the promoter regions of Kr-h1, G13402, 4EBP were 3.4, 2.6, 2.5 after JH III treatment and 2.7, 1.7, 2.5 after TSA treatment respectively when compared to DMSO treated cells. Moreover, when these cells were treated with dsCBP, it caused a significantly lower enrichment at the promoter region of these genes than in the control cells exposed to dsmalE (*P* < 0.0001). There is no significant enrichment at the promoter region of HSP90 in JH III and TSA treated TcA cells when compared with the DMSO treated cells (*P* > 0.05). These data suggest that CBP mediated acetylation of H3K27 plays a role in JH III or TSA induction of 4EBP, G13402, and Kr-h1 genes.

**Table 2:** F and P values in two way ANOVA analysis for chip assay.

	HSP90		Kr-h1		G13402		4EBP	
treatment	F value	P value	F value	P value	F value	P value	F value	P value
dsRNA	1.99	0.175	620.69	<0.0001	27.07	<0.001	78.6	<0.001
JHIII/TSA	3.56	0.052	362.37	<0.0001	11.19	<0.001	62.98	<0.001

## Discussion

The CBP is known to promote transcription through chromatin remodeling, acetylation of proteins, and recruitment of the basal transcription machinery^33,35,42–44^. In this study, we investigated the role of this multifunctional protein in JH action. JH is a pleiotropic hormone and regulates almost every aspect of insect’s life. In addition, the same circulating hormone is able to regulate many physiological processes differentially in a spatial and temporal fashion. Because of the complex nature of gene regulation by this hormone, simple receptor-ligand models may not be sufficient to explain multiple gene expression controls exerted by JH. Other layers of regulation of gene expression including epigenetics and post-translational modification of proteins may play important roles in the modulation of JH-response in a spatial and temporal manner. Recent studies identified bHLH transcription factor, Met as a JH receptor and steroid receptor co-activator, SRC/FISC/Taiman as a coactivator involved in JH action^1^. Since Met is the first bHLH-PAS protein identified as a receptor for the natural endogenous hormone, not much is known about the molecular mechanisms of Met action in transduction of JH signals.

In the current study, using well-functioning RNAi in *T. castaneum*, we showed that CBP is required for JH-induced Kr-h1, 4EBP and G13402 expression in TcA cells, *T. castaneum* larvae and adults (Figs 1–3). In addition, the expression of these three JH-response genes, Kr-h1, 4EBP, and G13402, is also induced by HDAC inhibitor, TSA (Fig. 4). Based on these data, we hypothesize that CBP is involved in JH induction of gene expression through acetylation of histones. Indeed, CBP mediated acetylation plays a major role in hormone-induced gene expression. In *D. melanogaster*, CBP functions as a histone acetyltransferase (HAT) in the 20E regulation of dendrite pruning through acetylation of H3K27 and CBP also physically interact with EcR, USP, and Brahma^28^. Histone acetyltransferase activity is also involved in the 20E-induced expression of Eip74EF and Eip75B, and CBP mediated acetylation of histone H3K23 is required for this action^29^. In mouse, CBP- and HDAC-mediated histone acetylation is a highly dynamic posttranslational modification, and this acetylation is required for progesterone receptor (PR)-mediated activation of the mouse mammary tumor virus promoter^45^. However, in the same cells, CBP recruitment increased histone acetylation, but this is not required for steroid induction of all genes^42^. Our studies also showed that CBP is required for induction of three JH-response genes. However, CBP is not required for induction of hsp90 gene.

**Figure 8:**
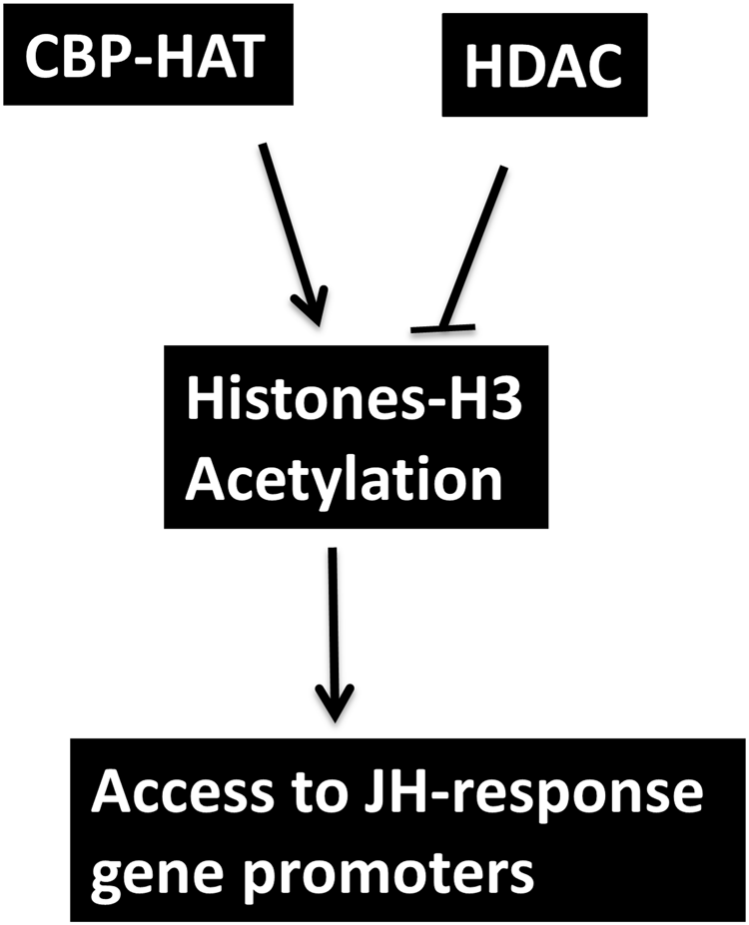
A model for CBP and HDAC regulation of acetylation and JH action.

Our recent studies in *T*. *castaneum* employing RNAi and RNA sequencing identified many target genes for CBP; some of these genes code for epifactor domains containing proteins^46^ Moreover, the regulation of JH response genes by CBP is also mediated by histone acetylation suggesting the epigenetic role for CBP in JH signaling. In chip assays, CBP knockdown affected the acetylation status of the H3k27 and thus reduced the enrichment of JH response genes (Kr-h1, 4EBP, G13402) promoters in the CBP knockdown samples. TSA (HDAC inhibitor) positively influenced the acetylation status of H3k27 by inhibiting the histone deacetylases (HDACs) resulting in enrichment of JH response gene promoters. Thus, it is likely that the JH response gene expression is regulated epigenetically through histone modification by CBP and HDACs (Fig. 8). The main contribution of the current study is the identification of role of histone acetylation in JH signaling. Even though the regulation of Kr-h1 is well studied in the insects^12,14,15,18–20^, none of these experiments addressed epigenetic regulation. Here, we report that the mRNA level of eukaryotic translation initiation factor (eIF) subunit 4E-binding protein (4EBP) gene, one of the key mTOR signaling components, is regulated by the JH signaling and CBP (Figs 1–4). Indeed, 4EBP was shown to be regulated by RPD3 (a conserved histone deacetylase) in the Drosophila^47^. However, further studies on the epigenetic regulation of JH action especially the role of HATs and HDACs as well as mechanisms underlying the function of CBP as an epigenetic regulator and coactivator are required to help in understanding the precise role of epigenetics in JH action.

## Materials and Methods

### Cell culture, constructs, and transfection

The *Tribolium castaneum* cells (BCIRL-TcA-CLG1) were grown at 28 °C in Ex-cell 420 (Sigma), containing 10% Fetal Bovine Serum (Gibco)^40^. TcA cells were seeded in 6-well plates. After overnight culture in the medium containing 10% FBS, the cells were treated with JH III or TSA dissolved in DMSO; 20E dissolved in distilled water. DMSO was added to the control cells. The DMSO concentrations were maintained at less than 0.1% level. At the end of the exposure period, the cells were rinsed with PBS, harvested and processed for isolation of RNA.

### dsRNA injection and topical application of JH analog hydroprene

The newly emerged male adults (within 6 hr after emergence) or final instar larvae were anesthetized with ether vapor for 4–5 min and lined on a glass slide covered with 2-sided tape. The dsRNA was injected into the dorsal side of the first or second abdominal segment using an injection needle pulled from a glass capillary tube using a needle puller (Idaho technology). About 0.8–1 µg (0.1 µl) dsRNA was injected into each new male adult or each larva. The *malE* dsRNA was used as a control. The injected beetles were removed from the slide and reared on whole-wheat flour at 30 ± 1 °C for 72 hr. For hormone treatment, hydroprene was topically applied to adults and larvae after 72 hr exposure to dsRNA. The animals were collected at 6 hr after treated with 10 µm hydroprene. Six to ten animals were used for each replicate, and three to four replicates were used for each treatment.

Total RNA was isolated using the TRI reagent (Molecular Research Center Inc., Cincinnati, OH). The DNA was eliminated from the total RNA using DNase I (Ambion Inc., Austin, TX) and 1 µg of total RNA for each sample was used for cDNA synthesis. cDNA as a template was used to amplify a fragment of the target gene, and the PCR product was used for dsRNA synthesis. Primers used for dsRNA synthesis and real-time PCR are listed in Table 1 or reported in our previous publication^6^. The MEGA script RNAi Kit (Ambion Inc., Austin, TX) was employed for dsRNA synthesis. For annealing dsRNA, the reaction mixture was incubated at 75 °C for 5 min and cooled it to room temperature over a period of 60 min. After the treatment with DNase I, dsRNA was purified by phenol/chloroform extraction followed by ethanol precipitation and the dsRNA concentration was determined using a NanoDrop 2000 spectrophotometer (Thermo Fisher Scientific Inc., Waltham, MA).

**Table 1:** Primers used in the paper.

**Gene Name**		**Primer sequence(5′-3′)**
**qRT-PCR primers**		
HSP90	F	CCAAGGCAAATTGCTCGACACGAT
	R	AGTCGTCCTTGCTCGGGTTATTGT
4EBP	F	GCGCTCCAACTACAAAGCGTTCAA
	R	TCCATGATGATCCCGTTCGTCCAA
CBP	F	TACCGCTCCAGTGATAATACGCCA
	R	TGGTAGCATTGACGCTTCTCGTCT
G13402	F	GCAACTTTGACAATGTCGTCGCCT
	R	ACCCACCCATAAGCCATTACCGTT
HR4	F	AACGATAACCTTGGGCGATTA
	R	CGATCATAATTTGGACGCTCTTG
E75A	F	GAAATCGCGTCCAAGTG
	R	GAAGGAAGTTCAATGGC
E75B	F	ATGCAGACCGCCACCATCG
	R	CGGGGATGGAGCTGGAGG
E74	F	GCGTCTTCAAGCTGGTG
	R	CAGCCTTTGACCGTCCAC
FTZ-F1	F	TGCGAGGAATCACAAACAAG
	R	TGTGACGTTTGCTCGAAGAC
Rp49	F	TGACCGTTATGGCAAACTCA
	R	TAGCATGTGCTTCGTTTTGG
**dsRNA primers**		
CBP	F	ACCGGGATTCTGGACTTTGATGGT
	R	AGCCCAGAATGACGATTGACTGGA
**Chip Assay**		
Kr-h1	F	GGCTGCAGACGACTTTCTTTA
	R	GCCGGAAATGGTCGGTTATTA
G13402	F	GACTGTCATCCACAGGGAAA
	R	CAAGGTGTCCTGCAACATTATC
4EBP	F	ACTGCCGATGCAAGTCAA
	R	CAGTACGAACGGAGACCATAAC
HR4	F	AACGATAACCTTGGGCGATTA
	R	CGATCATAATTTGGACGCTCTTG
hsp90	F	GCGCTAAGTGAAGAGCTAAGA
	R	ATGCACACACGAACAAATCAC

### Western blots

For Western blot hybridization, 50 µg total protein extracted from the day 5 adults and TcA cells were separated by SDS-PAGE gels. The proteins were transferred to the membrane and the membrane was cut into two parts. The lower part probed with acetyl-lysine, H3K9, H3K18 and H3K27 antibodies as described previously^4^. The upper blots probed with Actin antibody as a loading control.

### Chromatin Immunoprecipitation Assay

Chromatin Immunoprecipitation (ChIP) assay was performed using the TcA cells (2 × 10^6^ cells/assay), H3K27ac antibody and the Pierce Magnetic Chip Kit (Thermo Scientific) according to the manufacturer protocol. Briefly, TcA cells were incubated with 1 µg of malE or CBP dsRNA for 72 hr; then the cells were exposed to DMSO, 10 µm JH III or 5 µm TSA for an additional 6 hr. The cells were fixed with formaldehyde and subjected to overnight immunoprecipitation using H3K27ac antibody. The enrichment at the promoter region of three JH-response genes, Kr-h1, 4EBP, and G13402, were quantified by qPCR using specific primers (Table 1). As a control, the enrichment of the promoter region of HSP90 (housekeeping gene) was measured. Chip assay with IgG antibody served as a negative control for the experiment. The enrichments were represented as percent input for all the tested JH response genes. Chip assay experiment was repeated twice with similar results.

### Statistical analysis

All the statistical analysis is performed using SPSS 16.0. The gene expression data has been analyzed using one-way ANOVA or T-test. The Chip assay data were analyzed using two way ANOVA.

### Data Availability

The datasets generated during and/or analyzed during the current study are available from the corresponding author on reasonable request.

## Electronic supplementary material

Supplementary Information
