# Author Correction: CREB-binding protein plays key roles in juvenile hormone action in the red flour beetle, *Tribolium Castaneum*

**DOI:** 10.1038/s41598-018-30083-8

**Published:** 2018-08-02

**Authors:** Jingjing Xu, Amit Roy, Subba Reddy Palli

**Affiliations:** 1 Department of Entomology, College of Agriculture, Food and Environment University of Kentucky, Lexington, KY 40546 USA; 2 Faculty of Forestry and Wood Sciences, EXTEMIT-K, Czech University of Life Sciences, Kamýcká 1176, Prague 6, Suchdol, 165 21 Czech Republic

Correction to: *Scientific Reports* 10.1038/s41598-018-19667-6, published online 23 January 2018

This Article contains errors in the primer sequences reported in Table 1. The correct Table 1 appears below.

**Table 1:** Primers used in the paper.

Gene Name		Primer sequence (5′-3′)
qRT-PCR primers		
HSP90	F	GCGCTAAGTGAAGAGCTAAGA
	R	ATGCACACACGAACAAATCAC
4EBP	F	ATCACCGATGGCAAGACAAGTGAC
	R	ATGGCAGTTCAGAAGGGTCGTTGA
CBP	F	GGTCCCGATGGTAAGAAGAAAG
	R	CCGAGAGATCATTACCCGTTTG
G13402	F	ACTGTGCCGAGTTTAGGA
	R	GAATGCCAGTGGGTCAGG
HR4	F	ATAGAACAGCTCCGGCAAGA
	R	TGCCGCAGATTGTATCTCTG
E75A	F	GAAATCGCGTCCAAGTG
	R	GAAGGAAGTTCAATGGC
E75B	F	ATGCAGACCGCCACCATCG
	R	CGGGGATGGAGCTGGAGG
E74	F	GCGTCTTCAAGCTGGTG
	R	CAGCCTTTGACCGTCCAC
FTZ-F1	F	TGCGAGGAATCACAAACAAG
	R	TGTGACGTTTGCTCGAAGAC
Rp49	F	TGACCGTTATGGCAAACTCA
	R	TAGCATGTGCTTCGTTTTGG
dsRNA primers		
CBP	F	CCTCAGACTCCTCAGCTTACTA
	R	GAGCTGAAGTTGGCGTAAATTG
Chip Assay		
Kr-h1	F	GGCTGCAGACGACTTTCTTTA
	R	GCCGGAAATGGTCGGTTATTA
G13402	F	GACTGTCATCCACAGGGAAA
	R	CAAGGTGTCCTGCAACATTATC
4EBP	F	ACTGCCGATGCAAGTCAA
	R	CAGTACGAACGGAGACCATAAC
HR4	F	AACGATAACCTTGGGCGATTA
	R	CGATCATAATTTGGACGCTCTTG
hsp90	F	GCGCTAAGTGAAGAGCTAAGA
	R	ATGCACACACGAACAAATCAC

