# Supplementary material for: CREB-binding protein plays key roles in juvenile hormone action in the red flour beetle, Tribolium Castaneum
Source: Sci Rep. 2018 Jan 23;8:1426. doi: 10.1038/s41598-018-19667-6 (PMC5780420; doi:10.1038/s41598-018-19667-6)

CREB-binding protein plays key roles in juvenile hormone action in the red flour beetle,

Tribolium Castaneum

Jingjing Xu, Amit Roy and Subba Reddy Palli

Supplementary Figure 1: 50 µg total proteins extracted from the day 5 adults and TcA cells were separated by SDS-PAGE gels, transferred to the membrane and probed with acetyl lysine, H3K9, H3K18 and H3K27 antibodies. The same blots probed with actin antibodies are shown on the top. The full-length blots are shown and the cropped regions of these blots have been used to assemble Figure 6.

Fig.1S

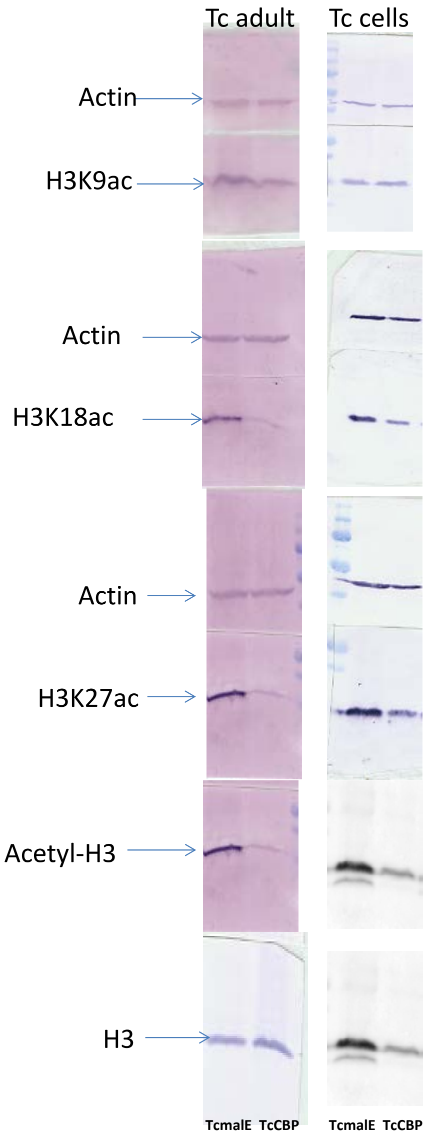

Supplement: Supplementary file 1 — Supplementary Information [file 41598_2018_19667_MOESM1_ESM.pdf]
